# Editorial Notes

**Published:** 1893-02

**Authors:** 


### Editorial Notes.

WE are in receipt of the Columbia cycling calendar for 1893.

WE are glad to note the recovery of Dr. H. F. Harris, after being confined to his room for some weeks.

DR. J. C. AVARY, after a short illness, has gone to Florida for a much-needed rest. He will be absent about ten days.

DR. M. B. HUTCHINS, the well known specialist in skin diseases, has decided also to take up genito-urinary and venereal diseases.

THE highest court of Germany has decided that human life dates from the beginning of labor, and that its destruction before full term is not murder.

M. HAFFKINS, of the Pasteur Institute, intends leaving shortly for India where he expects to apply his method to practical use in the regions of endemic cholera in British India and in Siam.

THE French Benevolent Society, of San Francisco, are going to erect a magnificent charity hospital in that city, costing $200,000. It will be built in Pavillions, and will accommodate 150 patients.

WINTERNITZ, of Vienna, has recently treated, with great successs, several cases of renal disease with the extract of the wild filberry. Weil, of Berlin, claims that it has apparently cured a case of diabetes mellitus.

THE eleventh annual issue of the International Medical Annual, published by E. B. Treat, New York, will be ready in a short while, and is announced as being a very complete work, edited by a corps of thirty-six distinguished collaborators widely known in both Europe and America.

THE newly-elected Superintendent of the Grady Hospital, Dr. Kennan, assumed the duties of his office January 1st.

Dr. C. J. George, the Superintendent *pro tem*, resumed his position as junior house physician, Dr. Nolan becoming head physician *vice* Dr. H. L. Gill, whose term of service had expired, and who has entered upon the practice of his profession in Atlanta.

---

THE Sanitarium of Dr. J. B. S. Holmes, at Rome, was totally destroyed by fire on the morning of January 27th. Loss, $100,000; insurance about $65,000. Many patients were in the building when the fire was discovered, but all were removed. Dr. Holmes had built up an excellent and flourishing institution at Rome, and we much regret his misfortune. He will continue his work, and has already arranged for suitable buildings in which to accommodate his patients.

---

AT the second annual meeting of the American Electro-Therapeutic Association, the following officers were elected for the ensuing year; President, Dr. Augustin H. Goelet, New York; 1st Vice-President, Dr. Wm. F. Hutchison, Providence, R. I.; 2d Vice-President, Dr. W. J. Herdman, Ann Arbor, Michigan.; Secretary, Dr. Margaret A. Cleaves, New York; Treasurer, Dr. R. J. Nunn, Savannah, Ga. The next meeting will be held in Chicago on Sept. 12th, 13th, and 14th, 1893.

---

ONE of the series of International Congress to be held in Chicago in 1893 is to be devoted to the subjects of Charities, Correction and Philanthrophy, and the Fourth Section of this is to consider all matters relating to the Hospital Care of the Sick, The Training of Nurses, Dispensary Work, and First Aid to the Injured. The Committee of Organization of the Congress has appointed Dr. John S. Billings, Surgeon U. S. Army, as Chairman of this Section, and Dr. Henry M. Hurd, Superintendent of the Johns Hopkins Hospital, as its Secretary, and has authorized and requested them to complete its organization, to extend invitations and to prepare a programme for the meeting.

ONE of the rules governing the World's Columbian Exposition reads as follows: "Articles that are in any way dangerous or offensive, also patent medicines, nostrums, and impirical preparations whose ingredients are concealed, will not be admitted to the Exposition."

---

We desire to call the attention of our readers to the reply of Chas. Marchand to the attack of Dr. Jacobi, of New York, upon his preparation of proxide of hydrogen. Marchand's preparation of this drug has always been considered by us a most excellent one, and the assault of Dr. Jacobi seems altogether unwarranted.

---

THE following was sent to an Atlanta doctor with a specimen of urine in a half-pint flask for examination:

My morning "extract" here I send,
'Tis what you wanted, I suppose;
I hope and trust 'twill not offend,
Or make you elevate your nose.

No cocktail sweet, no toddy long,
This festive vessel doth contain,
And though it is not very strong,
I'm sure that it would stand a strain.

Although 'tis pleasant to the sight,
I send it with an anxious sigh,
And hope that no disease of Bright
Will meet your penetrating eye.

Dear Doctor, note the amber gleam,
But do not be deceived by this;
Things are not always what they seem,
This half-pint flask is full of ——!

